# Role of risk factors, scoring systems, and prognostic models in predicting the functional outcome in meningioma surgery: multicentric study of 552 skull base meningiomas

**DOI:** 10.1007/s10143-023-02004-5

**Published:** 2023-05-23

**Authors:** Michaela May, Vojtech Sedlak, Ladislav Pecen, Vladimir Priban, Pavel Buchvald, Jiri Fiedler, Miroslav Vaverka, Radim Lipina, Stefan Reguli, Jozef Malik, David Netuka, Vladimir Benes

**Affiliations:** 1 Department of Neurosurgery and Neurooncology, First Faculty of Medicine, Charles University and Military University Hospital, U Vojenske nemocnice 1200, 16902 Prague, Czech Republic; 2 First Faculty of Medicine, Charles University in Prague, Prague, Czech Republic; 3 Department of Radiology, Military University Hospital, Prague, Czech Republic; 4 Institute of Computer Science, The Czech Academy of Sciences, Prague, Czech Republic; 5 Department of Neurosurgery, Pilsen University Hospital, Pilsen, Czech Republic; 6 Department of Neurosurgery, Liberec Hospital, Liberec, Czech Republic; 7 Department of Neurosurgery, Ceske Budejovice Hospital, Ceske Budejovice, Czech Republic; 8 Department of Neurosurgery, University Hospital Olomouc, Olomouc, Czech Republic; 9 Department of Neurosurgery, University Hospital Ostrava, Ostrava, Czech Republic

**Keywords:** Meningioma, Skull base, Surgery, Outcomes, Karnofsky Performance Status Scale, Predictive factors

## Abstract

Despite the importance of functional outcome, only a few scoring systems exist to predict neurologic outcome in meningioma surgery. Therefore, our study aims to identify preoperative risk factors and develop the receiver operating characteristics (ROC) models estimating the risk of a new postoperative neurologic deficit and a decrease in Karnofsky performance status (KPS). A multicentric study was conducted in a cohort of 552 consecutive patients with skull base meningiomas who underwent surgical resection from 2014 to 2019. Data were gathered from clinical, surgical, and pathology records as well as radiological diagnostics. The preoperative predictive factors of functional outcome (neurologic deficit, decrease in KPS) were analyzed in univariate and multivariate stepwise selection analyses. Permanent neurologic deficits were present in 73 (13.2%) patients and a postoperative decrease in KPS in 84 (15.2%). Surgery-related mortality was 1.3%. A ROC model was developed to estimate the probability of a new neurologic deficit (area 0.74; SE 0.0284; 95% Wald confidence limits (0.69; 0.80)) based on meningioma location and diameter. Consequently, a ROC model was developed to predict the probability of a postoperative decrease in KPS (area 0.80; SE 0.0289; 95% Wald confidence limits (0.74; 0.85)) based on the patient’s age, meningioma location, diameter, presence of hyperostosis, and dural tail. To ensure an evidence-based therapeutic approach, treatment should be founded on known risk factors, scoring systems, and predictive models. We propose ROC models predicting the functional outcome of skull base meningioma resection based on the age of the patient, meningioma size, and location and the presence of hyperostosis and dural tail.

**Supplementary Information:**

The online version contains supplementary material available at 10.1007/s10143-023-02004-5.

## Introduction

Meningiomas are the most frequent primary intracranial and central nervous system tumors [1]. Treatment modalities consist of observation, surgical resection, stereotactic radiosurgery (SRS), fractionated external beam radiotherapy (EBRT), and pharmacotherapy [2]. Shortly, the therapeutic approach will be refined by recent advances in molecular genetics [2]. In 2017, Sahm et al. introduced DNA methylation-based classification, which has high power to predict meningioma prognosis and recurrence [3]. Additionally, molecular markers as grading criteria for selected meningioma subtypes were introduced by the 2021 World Health Organization **(**WHO) classification [4].

Although recent advances in molecular genetics enable better patient stratification, surgical decision-making is based on preoperative predictive factors in a patient with newly diagnosed meningioma [3, 4]. For symptomatic or progressive meningiomas, the first-line treatment option in contemporary practice is a maximal safe resection [2]. In a recent series, the reported rates of neurologic morbidity and mortality are 3.9–13.7% and 0–5.4%, respectively [5–7]. Meling et al. documented a significantly higher risk of postoperative neurologic deterioration (21 vs. 13%) in skull base meningiomas (SBMs) compared to non-skull base meningiomas (nSBMs) [8]. This risk is especially apparent in SBMs because of their close relationship to critical neurovascular structures [9].

Despite the importance of the functional outcome, only several studies have focused on the prognostic factors in meningioma surgery [5, 7, 8, 10–15]. Moreover, only a few scoring systems exist to predict neurologic outcome [6, 16–19]. Our study aims to identify preoperative factors predicting the functional outcome of SBM resection. Thus, based on the risk factors identified, the ROC models estimating the risk of a new postoperative neurologic deficit and a decrease in KPS are developed and compared to the existing scoring systems in the literature review.

## Material and methods

In this multicentric study 552 consecutive patients who underwent SBM resection from January 1, 2014 to December 31, 2019 were investigated. The data were collected retrospectively from January 1, 2014 to June 30, 2018 and prospectively from July 1, 2018 to December 31, 2019. Our analysis included data from six neurosurgical centers in the Czech Republic (Military University Hospital Prague, 260 patients; Pilsen University Hospital, 76 patients; Liberec Hospital, 69 patients; Ceske Budejovice Hospital, 63 patients; University Hospital Olomouc, 44 patients; and University Hospital Ostrava, 40 patients). Data were gathered from clinical, surgical, and pathology records as well as radiological diagnostic (magnetic resonance imaging [MRI] scans, computed tomography scans, digital subtraction angiography); subsequently, all data were anonymized. Radiological findings were evaluated by two independent senior radiologists (VS and JM). The degree of resection in the surgical records was estimated using the Simpson grading system. However, the extent of resection was consequently verified on early baseline postoperative MRI. Clinical and radiological controls were performed according to protocols of individual departments, but at least regularly once a year.

The following information was included in the database:

- General characteristics: patient age, sex, date of birth, date of resection, and follow-up duration.
- Preoperative status: symptoms (patient’s subjective perceptions suggesting bodily defect or malfunction), signs (objective indications of disease) and their duration (days, months etc.), preoperative Glasgow Coma Scale (GCS), [20] and the Karnofsky performance status (KPS) [21].
- Radiological characteristics: meningioma diameter, dimensions (a, b, c), volume, location, shape, margins, tumor-brain interface, presence of arachnoid plane, arachnoid cistern of SBM origin, edema, enhancement, capsular enhancement, dural tail, vessel encasement/narrowing, cavernous sinus invasion, cysts, sunburst sign, bone invasion, and hyperostosis (Table 1).
- Surgery: surgical approach, Simpson grade of resection, [22] complications, and surgical revisions.
- Histopathological analysis: WHO grade. Histopathological diagnoses were performed using the 2007 and 2016 WHO classifications [23, 24].
- Clinical outcome: evolution of preoperative symptoms and signs (improved, stable, worsened), new neurologic deficits (temporary or permanent, evaluated 1 year after surgical resection), KPS, and the Glasgow Outcome Scale (GOS) [25].
- Radiological outcome: tumor progression date and recurrence date.
- Further therapeutical management: SRS, EBRT, and surgical resection.

**Table 1:** Radiologic characteristics—evaluation of MRI characteristics

Radiologic characteristics	
Diameter	Mm
Volume	*V* = 4/3 × *π* × *a*/2 × *b*/2 × *c*/2; cm^3^ *a*, *b*, *c* – SBM dimensions in axial, coronal, and sagittal planes
Location	Supratentorial and infratentorial
	Olfactory groove, planum sphenoidale, tuberculum sellae, sella turcica, sphenoid wing - medial, middle and lateral variant, sphenoorbital, frontobasal, cavernous sinus, middle cranial fossa, Meckel´s cave, posterior clinoid process, petrous, petroclival, clival, cerebellopontine angle, jugular foramen and foramen magnum
	Olfactory, carotid, chiasmatic, ambient, interpeduncular, prepontine, premedullary, cerebellopontine, cerebellomedullary, and unassignable position of the SBM origin within the arachnoid cisterns
Vessel encasement	Absent: no contact; partial: < 360° encasement; complete: 360° encasement Assessed on proximal arteries of the circle of Willis: ICA and MCA (M1 and M2 segments), ACA (A1 and A2 segments), VA, BA, ACOM, and PCOM.
Vessel narrowing	Absent: no effect on vessel lumen; present: narrowing compared to distal segments of the same artery or narrowing of the vessel compared to the contralateral side without other possible explanations (variant/asymmetric COW, etc.) T1-weighted gadolinium-enhanced images, T2-weighted imaging
Cavernous sinus invasion	Absent: normal appearance; present: asymmetry of the cavernous sinus T1-weighted gadolinium-enhanced images, T2-weighted imaging
Shape	Regular: ellipsoidal/semielipsoidal shape; irregular: polycyclic appearance, indentations, and sessile growth
Invasive tumor-brain interface	Absent, present present if no clear well-defined meningioma margin is visible T1-weighted gadolinium-enhanced images, T2-weighted imaging
Margins	Smooth, irregular (mushroom-like growth, nodularity, etc.)
Enhancement	Homogenous, heterogenous, and faint T1-weighted gadolinium-enhanced images
Capsular enhancement	Absent, present (greater than half of the tumor surface enhanced) T1-weighted gadolinium-enhanced images
Dural tail	Absent, present T1-weighted gadolinium-enhanced images
Edema	Absent; less than the meningioma diameter; greater than the meningioma diameter T2-weighted imaging
Arachnoid plane	Absent, present presence of a cerebrospinal fluid cleft at the meningioma surface T2-weighted imaging
Cysts	Absent, present (intra- or peritumoral cysts)
Sunburst sign	Absent, present
Bone invasion	Absent, present
Hypeostosis	Absent, present

### Outcome measures

We further defined functional outcome as favorable (absence of a new neurologic deficit, increased or unchanged KPS) or unfavorable (presence of a new neurologic deficit, decrease of KPS ≥10). Predictive factors of unfavorable clinical outcome were analyzed.

### Literature review

To summarize known risk factors, surgical risk scales and grading systems for functional outcome in meningioma (or only SBM) surgery a PubMed search were performed for entries until July 3, 2022, using the following query guidelines: 1) (meningioma) AND (grading system) AND (outcome) with 237 results; 2) (meningioma) AND (surgical scale) with 418 results; and 3) (meningioma) AND (risk factors) AND ((functional outcome) OR (neurologic outcome) OR (clinical outcome)) with 330 results. Case reports, non-English studies, conference papers, and abstracts were not included. Exclusion criteria were selective anatomical location and histological meningioma variants, patient subgroup (e.g., elderly), and extracranial meningioma location. From a search of other relevant resources, the grading system CLASS algorithm was included (Lee et al.) [6]. The Milan Complexity Scale was included in the review because of its importance, although other brain tumors were included [18].

### Statistical analysis

Baseline data are presented descriptively as means and standard deviations (SDs) for normal distributions, median, and interquartile range for non-normally distributed data and absolute and relative frequencies for qualitative variables as summary statistics. Inferential statistical analysis was done using logistic regression (univariate and multivariate stepwise selections). Group comparisons were performed employing Wilcoxon rank sum tests and the Kruskal–Wallis test for more than two groups. The relationship between numerical parameters was investigated by correlation analysis using the Spearman rank correlation coefficient and chi-square test for qualitative variables. All inferential statistics were presented with appropriate 95% confidence intervals and reported along with their *p*-values. SAS version 9.4 (SAS Institute Inc., Cary, NC, USA) software was used for all statistical analyses. For all hypotheses tested, a *p*-value < 0.05 indicated statistical significance. All tests were performed as two sided. No adjustment for multiple comparisons was made because there is no single primary hypothesis.

### Ethics approval

All procedures performed in studies involving human participants were in accordance with the ethical standards of the Ethical Committee of University Hospital in Ostrava (reference number 530/2018) and with the 1964 Helsinki Declaration and its later amendments or comparable ethical standards.

## Results

From January 1, 2014 to December 31, 2019, 552 consecutive patients underwent surgical resection for SBMs. The cohort contained 423 women (76.6%) and 129 men (23.4%). The mean age of patients at surgery was 56.8 (range 20–85, median 58) years. The mean preoperative KPS was 90 (median 90). Objective neurological deficits were documented in 355 patients (64.3%). The average duration of clinical signs or symptoms was 56 months. Radiological characteristics are outlined in Table 2. Some 452 meningiomas (81.9%) were supratentorial and 100 (18.1%) infratentorial. The average diameter was 3.1 cm and the average volume was 22.7 cm^3^. The extent of resection followed Simpson grade (S) I in 87 (16.9%), SII in 321 (58.2%), SIII in 34 (6.2%), SIV in 109 (19.7%), and SV in 1 (0.2%) patient. Histological analysis revealed grade 1 meningiomas in 511 (92.6%) and grade 2 in 41 (7.4%) cases.

**Table 2:** Radiologic characteristics

Characteristic	No	%	Location	No	%
Irregular shape	98	17.8	Olfactory groove	63	11.4
Invasive tumor-brain interface	79	14.3	Planum sphenoidale	58	10.5
Irregular margins	131	23.7	Tuberculum sellae	55	10.0
Arachnoid plane	213	38.6	Sella turcica	3	0.5
Peritumoral edema	236	42.8	Sphenoorbital	39	7.1
Contrast enhancement—homogeneous	443	80.3	Sphenoid wing, medial variant	84	15.2
Contrast enhancement—heterogeneous	108	19.6	Sphenoid wing, middle variant	39	7.1
Contrast enhancement—faint	1	0.2	Sphenoid wing, lateral variant	43	7.8
Capsular enhancement	85	15.4	Frontobasal	15	2.7
Dural tail	323	58.5	Cavernous sinus	9	1.6
Major vessel—contact	234	42.4	Middle cranial fossa	28	5.1
Major vessel—360° encasement	87	15.8	Posterior clinoid process	8	1.4
Major vessel—narrowing	9	1.6	Petrous	30	5.4
Cavernous sinus invasion	36	6.5	Petroclival	14	2.5
Sunburst sign	179	32.4	Clival	5	0.9
Intra- or peritumoral cysts	54	9.8	Cerebellopontine angle	43	7.8
Bone invasion	129	23.4	Jugular foramen	3	0.5
Hyperostosis	102	18.5	Foramen magnum	13	2.4

For statistical analysis, due to the limited number of patients with SBM in rare locations, these locations were considered together with meningiomas in adjacent locations: tuberculum sellae with sella turcica, posterior clinoid process with petrous, petroclival with clival, and jugular foramen with cerebellopontine angle meningiomas (Table 3).

**Table 3:** Meningioma locations for the statistical analysis

Location	No	%
Olfactory groove	63	11.41
Middle cranial fossa	28	5.07
Petrous + posterior clinoid process	38	6.88
Petroclival + clival	19	3.44
Cerebellopontine angle + jugular foramen	46	8.33
Foramen magnum	13	2.36
Sphenoorbital	39	7.07
Planum sphenoidale	58	10.51
Sella turcica + tuberculum sellae	58	10.51
Sphenoid wing, medial variant	84	15.22
Sphenoid wing, middle variant	39	7.07
Sphenoid wing, lateral variant	43	7.79
Frontobasal	15	2.72
Cavernous sinus	9	1.63

### Clinical outcome

Overall survival (OS) at 1 and 2 years was 98.1% (average follow-up 27.7 months). The distribution of GOS in the cohort of our patients was as follows: 5 in 436 (79.0%), 4 in 85 (15.4%), 3 in 16 (2.9%), 2 in 5 (0.9%), and 1 in 10 (1.8%) patients. Surgery-related mortality was present in seven cases (1.3%) and not surgery-related in three cases (0.5%). The mean KPS at discharge was 89 (median 90). The KPS remained unchanged or increased in 468 (84.8%) patients and decreased in 84 (15.2%). The neurologic deficit, present initially in 355 patients, improved in 158 (44.5%), remained unchanged in 159 (44.8%), and worsened in 38 (10.7%). Temporary and permanent neurologic deficits were observed in 57 (10.3%) and 73 (13.2%) patients, respectively. The temporary neurologic deficits were CN palsy in 27 (4.9%, the most common oculomotor nerve palsy in 16 patients; 2.9%), motor deficit in 10 (1.8%), speech disorder in 9 (1.6%), cognitive decline in 4 (0.7%), somatosensory deficit in 2 (0.4%), cerebellar signs in 2 (0.4%), and higher cortical function deterioration in 2 patients (0.4%). Epileptic seizure was recorded in 7 patients (1.3%). The permanent neurologic deficits were CN palsy in 52 (9.4%; the most common oculomotor nerve palsy in 48 patients; 8.7%), motor deficit in 12 (2.2%), cognitive decline in 7 (1.3%), speech disorder in 4 (0.7%), higher cortical function deficit in 2 (0.4%), cerebellar signs in 2 (0.4%), and somatosensory deficit in 2 (0.4%). Secondary epilepsy was present in 3 patients (0.5%).

### Predictive factors of clinical outcome

Predictive factors associated with a new neurologic deficit (temporary or permanent, evaluated at 1 year from surgical resection) and a decrease in KPS (at patient discharge) in univariate analysis are listed in Table 4.

**Table 4:** Univariate analysis—predictive factors of a new neurologic deficit and a postoperative decrease in KPS (chi, chi-square test; KW, Kruskal-Wallis test; W, Wilcoxon test)

New neurologic deficit	*p*		Decrease in KPS	*p*	
Location	Chi	< 0.0001	Diameter	W	0.0002
Major vessel—contact	Chi	< 0.0001	Volume	W	0.0002
Arachnoid cistern of origin	Chi	0.0002	Arachnoid cistern of origin	Chi	0.0004
Diameter	W	0.0047	Location	Chi	0.0008
Volume	W	0.0050	Edema	Chi	0.0022
Location (supra × infratentorial)	Chi	0.0065	Location (supra × infratentorial)	Chi	0.0026
Cavernous sinus invasion	Chi	0.0081	Hyperostosis	Chi	0.0093
Vessel narrowing	Chi	0.0225	Age	W	0.0106
Edema	Chi	0.0500	Major artery—contact	Chi	0.0118
			GCS	W	0.0311
			Capsular enhancement	Chi	0.0499

The predictive factors of a new neurologic deficit (temporary or permanent, evaluated at 1 year from surgical resection) and a decrease in KPS (at patient discharge) selected by the multivariate stepwise selection model are presented in Table 5.

**Table 5:** Multivariate stepwise selection analysis: predictive factors of a new neurologic deficit and a postoperative decrease in KPS (DF, degrees of freedom)

Summary of stepwise selection							
Step	Effect		DF	Number In	Score	Wald	*P*-value
	Entered	Removed			Chi square	Chi square	
New neurologic deficit							
1	Location		13	1	66.1644		< 0.0001
2	Diameter		1	2	8.1582		0.0043
3	Volume		1	3	4.1943		0.0406
4		Volume				3.7206	0.0537
Decrease in KPS							
1	Location		13	1	34.9892		0.0008
2	Diameter		1	2	21.6343		< 0.0001
3	Age		1	4	5.3024		0.0213
4	Dural tail		1	3	4.1730		0.0411
5	Hyperostosis		1	5	4.3647		0.0367
6	Volume		1	6	2.8577		0.0909
**7**		Volume	1	5		2.8223	0.0930

### New neurological deficit

According to univariate analysis (done by the logistic regression univariate model), the risk factors associated with higher probability of a new neurologic deficit were the following: presence of major vessel contact, higher diameter, higher volume, supratentorial location, presence of cavernous sinus invasion, presence of vessel narrowing, and presence of edema. Considering the location and arachnoid cistern of origin, there were statistically significant differences in the risk of new neurologic deficit among the subgroups.

In multivariate analysis (done by the multivariate stepwise selection logistic regression model), higher diameter (*p* = 0.0043) and higher volume (*p* = 0.0406) were associated with higher probability of a new neurologic deficit. Considering the location (*p* < 0.0001), the lowest probability of a new neurologic deficit was in cerebellopontine angle and jugular foramen meningioma and the highest probability in petroclival and clival meningiomas (in the list of locations* with its values—the higher the value is, the higher is the risk of a new neurologic deficit).

**Fig. 1:**
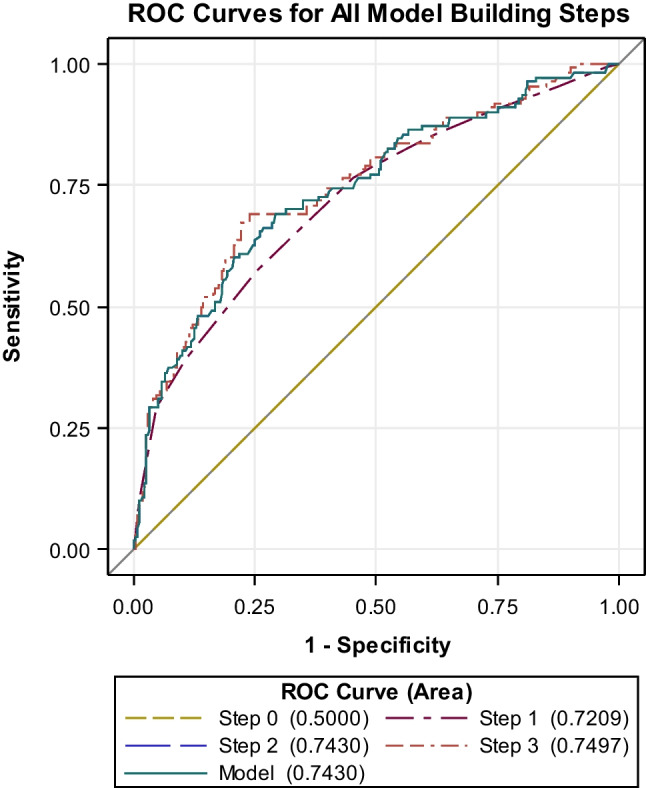
ROC model estimating the risk of a new neurologic deficit (area 0.74; 95% Wald confidence limits (0.69; 0.80))

To build a ROC model, parameter volume was excluded because of non-significance (Wald criterion). Based on the results, a ROC model estimating the risk of a new neurologic deficit was compiled (area under the receiver operating characteristic curve (area) 0.74; SE 0.0284; 95% Wald confidence limits (0.69; 0.80); Somers’ *D* 0.49; gamma 0.49; tau-a 0.18; Fig. 1). These results were converted to a simple excel calculator attached (calculator 1—risk of a new neurologic deficit).

**Table:** Risk score (below f (X)) = −2.1282 + 0.0289 × diameter (mm) + location ∗

*Location			
Cerebellopontine angle + jugular foramen	− 1.1002	Middle cranial fossa	0.2020
Sphenoid wing, lateral variant	− 0.9969	Sphenoid wing, medial variant	0.0000
Frontobasal	− 0.9789	Cavernous sinus	0.0571
Olfactory groove	− 0.7088	Sphenoid wing, middle variant	0.1872
Sella turcica + tuberculum sellae	− 0.4329	Foramen magnum	0.3805
Sphenoorbital	− 0.2768	Petrous + posterior clinoid process	1.7218
Planum sphenoidale	− 0.2290	Petroclival + clival	2.2125

The probability of a new neurologic deficit is then $y=\frac{1}{1+{e}^{-f(X)}}$

### Postoperative decrease in KPS

According to univariate analysis (done by the logistic regression univariate model), factors associated with higher probability of a decrease in KPS at patient discharge were higher diameter, higher volume, presence of edema, infratentorial location, higher age, major artery contact, and lower GCS. Presence of hyperostosis and presence of capsular enhancement were associated with lower probability of a decrease in KPS. Considering the location and arachnoid cistern of origin, there were statistically significant differences in the risk of a decrease in KPS among the subgroups (see below).

Factors associated with higher probability of a decrease in KPS in multivariate analysis (done by the multivariate stepwise selection logistic regression model) were higher diameter (*p* < 0.0001), higher volume (*p* = 0.0909), higher patient age (*p* = 0.0213), and presence of dural tail (*p* = 0.0411). Conversely, presence of hyperostosis (*p* = 0.0367) was a protective factor. Concerning the location (*p* = 0.0008), the lowest probability of a decrease in KPS was in frontobasal meningiomas and the highest in petrous and posterior clinoid process meningiomas (in the list of locations** with its values—the higher the value is, the higher is risk of decrease in KPS).

**Fig. 2:**
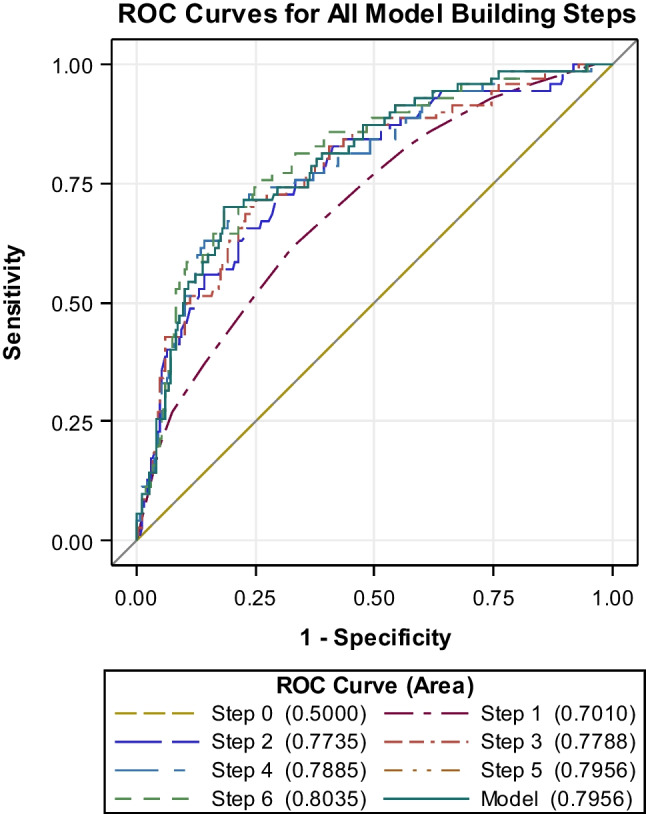
ROC model estimating the risk of a postoperative decrease in KPS (area 0.80; 95% Wald confidence limits (0.74; 0.85))

To build a ROC model, the volume parameter was discarded because of non-significance (Wald criterion). Based on the statistical results, a ROC model estimating the risk of a postoperative decrease in KPS was established (area 0.80; SE 0.0289; 95% Wald confidence limits (0.74; 0.85); Somers’ D 0.59; gamma 0.59; tau-a 0.16; Fig. 2). These results were converted to a simple excel calculator attached (calculator 2—risk of a decrease in KPS).

**Table:** Risks core (bellow f(X)) = − 6.2271 + 0.028 0 × age (years) + 0.0 514 × diameter (mm) + 0 .9781 x dural tail (0/1) − 1.3962 x hyperostosis(0/1) + location**

**Location			
Frontobasal	− 12.0078	Planum sphenoidale	0.4663
Sphenoid wing, lateral variant	− 1.1138	Cavernous sinus	0.9606
Olfactory groove	− 0.3455	Sella turcica + tuberculum sellae	1.7553
Middle cranial fossa	− 0.2336	Sphenoorbital	1.7941
Sphenoid wing, medial variant	0	Foramen magnum	1.8534
Sphenoid wing, middle variant	0.3612	Petroclival + clival	2.2055
Cerebellopontine angle + jugular foramen	0.3976	Petrous + posterior clinoid process	3.0063

The probability of a decrease in KPS is then $y=\frac{1}{1+{e}^{-f(X)}}$

Based on the literature search, four articles and one chapter met the selection criteria for the scoring system predicting clinical outcome. Nine articles met the criteria for detecting general risk factors predicting the functional outcome in meningioma surgery. The selected references and their basic characteristics are summarized in Table 6.

**Table 6:** Scoring systems and prognostic factors predicting the functional outcome in meningioma surgery—literature review

**Author, year, characteristics**	**Scoring system**						**Results and recommendations**
Levine et al [19]. 1999 **Levine-Sekhar grading system** **Locations:** SBM **Design:** retrospective **Cohort:** 232 patients **Years:** 1993–1997 **Outcome measure:** KPS (last follow-up) Length of hospital stay EOR	**Category**	**Variable**	**Presence**	**Absence**	**Score**		**Grade—total score—mean KPS change:** Grade 0: 0; 7 p. Grade I: 1,2; 9 p. Grade II: 3,4; 13 p. Grade III: 5,6; 20 p. Inverse linear correlation of grade and resection rate. The only significant predictor of better FU KPS was the preoperative KPS score. If variables combined as resection grade, lower resection grades were correlated with better FU KPS scores.
	**History**	Previous RT	1	0	0–1		
	**Imaging studies**	Vessel encasement	1	0	0–2		
		Multiple fossa involvement	1	0			
	**Physical examination**	CN III palsy	1	0	0–3		
		CN V palsy	1	0			
		CN VI palsy	1	0			
	**Total score**				0–6		
Lee and Sade et al. [6]. 2009 **CLASS algorithm** **Locations:** all **Design:** retrospective, prospective **Cohort:** 300 patients (+ 236 patients—evaluation) **Years:** 2000-2004 **Outcome measure**: GOS (at 6 weeks; 4 + 5—favorable) Neurological and medical complications	**Factors**	**Score**					**Group—total score—poor outcome—NC - MC (%):** Group 1: ≥ +1; 1.8%; 7.3%; 1.8% Surgery recommended. Group 2: 0, − 1; 3.9%; 15.6%; 6.5% Surgery is considered with caution. Group 3: ≤ − 2; 16.2%; 24.3%; 10.8% Surgery is not recommended.
		**− 2**	**− 1**	**0**	**1**	**2**	
	**Comorbidity**	ASA3	ASA2	ASA1			
	**Location**	Complex	Moderate	Simple			
	**Age**	≥ 71	61–71	≤ 60			
	**Size**			≤ 2 cm	2.1–4cm	> 4 cm	
	**Signs/symptoms**			Asympt.	+	++	
	**Others**		Prior RT/Sx		Progression		
Adachi et al [16]. 2009 **ABC Surgical Risk Scale** **Locations:** SBM **Design:** retrospective **Cohort:** 132 patients (+ 60 patients validation) **Years:** 2000–2005 (+ 1995–2000) **Outcome measure:** KPS EOR	**Factors**	**Points**					**Grade—total score—neurologic deterioration (%):** Grade I: 0–4; 7.8% Surgery recommended. Grade II: 5–7; 28.1% Surgery recommended. Grade III: 8–12; 46.7% STR followed by SRS recommended. In most GIs, GTR was achieved and NTR was achieved in 90% of GII; however, 100% of GIII were amenable only to NTR and STR. Weighting neurologic change against the EOR suggests that extensive surgery causes neurologic deterioration in GII and GIII cases.
		**0**	**1**	**2**			
	**Attachment size**	< 2 cm	2–4 cm	> 4 cm			
	**Arterial involvement**	None	Single	Multiple			
	**Brainstem contact**	CSF space visible	No CSF space visible	No CSF space visible with perifocal edema			
	**Central cavity**	Outside	Partial involvement	Inside			
	**CN group involvement**	0	1	≥ 2			
	**History of RT or Sx**	+ 1 point for each					
	**Total score**	0–12 points					
Bartek et al. [17]. 2015 **Predictors of severe complications** **Locations:** all **Design:** retrospective **Cohort:** 979 patients **Years:** 2007–2013 **Outcome measure:** Severe complications (Ibanez class. [26].)	**Factors**		**Points**				**Total score—risk of severe complication (Ibanez cl., %):** 0 p.: 2.2% (low risk) 1 p.: 7.6% (medium-low risk) 2 p.: 14.2% (medium-high risk) 3 p.: 46.7% (high risk)
	**Age > 70 years**		1				
	**KPS score < 70**		1				
	**Surgery duration > 4 h**		1				
	**Total score**		0–3				
*Ferroli et al. [18]. 2015 **Milan Complexity Scale** **Tumors:** meningiomas (28.6%), glioblastomas (24.1%), adenomas (8.4%), anaplastic astrocytomas (8.0%), etc. **Design:** retrospective **Cohort:** 746 **Years:** 2012–2014 **Outcome measure:** KPS	**Variable**	**0**	**1**	**2**	**3**		**Total score—risk of worsening (%):** 0 p.: 7.6% 1 p.: 13.9% 2 p.: 20.3% 3 p.: 44.4% 4 p.: 58.6% 5 p.: 72.4% 6-8 p.: 60% Scores higher than 3 are indicative of an increased risk of worsening. Evaluation of the five parameters mentioned above (the Big Five), the Milan Complexity Scale enables neurosurgeons to estimate the risk of a negative clinical course after brain tumor surgery.
	**Major vessel manipulation**	No	Yes				
	**Posterior fossa**	No	Yes				
	**CN manipulation**	No		Yes			
	**Eloquent area**	No			Yes		
	**Tumor size**	0–4 cm	≥ 4.1 cm				
	**Total score**	0–8 points					
**Author, year, characteristics**	**Predictive factors**						**Results and recommendations**
Meixensberger et al. [10]. 1996 **Locations:** all **Design**: retrospective **Cohort:** 385 patients **Years:** 1975–1988 **Outcome measure:** KPS (30 days, 6 months)	**Decrease of KPS:** Age Poor preoperative clinical condition (ASA score) Intra- and postoperative bleeding CSF disturbances Optic nerve and other CN disturbances Medial SWM **Increase of KPS:** Intracranial hypertension Seizures Aphasia Hemiparesis						Age, poor preoperative clinical condition (ASA score), intra- and postoperative bleeding, and CSF disturbances were significantly associated with a subsequent decrease in quality of life. First symptoms, such as intracranial hypertension, seizures, aphasia, and hemiparesis, were correlated with an increase in the postoperative Karnowsky index. Postoperative quality of life significantly decreased in patients with optic and other cranial nerve disturbances. Tumor size, location (exception: medial sphenoid wing) and histological diagnosis did not influence the surgical outcome.
Miao et al. [11]. 2010 **Locations:** all **Design:** retrospective **Cohort:** 147 patients **Years:** 2002–2004 **Outcome:** modified questionnaire based on the WHO QoL-100 Scale KPS	**UVA -** Health-related QoL: Tumor size EOR Histologic grade **MVA:** HQOL = 119.1097 – 1.5002*X*_3_ – 8.6650*X*_6_ – 10.4210*X*_7_ (*R* = 0.7466; where *X*_3_ is tumor size, *X*_6_ is EOR, and *X*_7_ is the histologic grade of the tumor)						This equation can be used preoperatively to predict the HQOL of meningioma patients after neurosurgery.
Scheitzach et al. [13]. 2014 **Locations:** SBM **Design:** retrospective **Cohort:** 226 patients **Outcome:** neurological improvement KPS Medical Research Council Neurological Severity Score (MRC-NPS) RR	The improvement of the MRC-NPS and KPS scores differed significantly with tumor location, with olfactory groove and lateral sphenoid wing tumors showing the best and foramen magnum meningiomas the worst functional results at follow-up. **Location improvement rate:** Medial sphenoid ridge 4.5 Olfactory groove 12.1 Petroclival 6.2 Tentorial 6.5 Tuberculum sellae 9.1 Foramen magnum 0.0 Lateral sphenoid ridge 62.5						The MRC-NPS and KPS significantly improved from the preoperative status to discharge; however, the improvement rate depended on the tumor location.
Zeng et al. [15]. 2015 **Locations:** all asymptomatic **Design:** retrospective **Cohort:** 513 patients **Years:** 2007–2012 **Outcome measure:** GOS	UVA and MVA: age < 60 years						The age of the patient and the location of the tumor should be carefully considered before choosing to perform surgery on asymptomatic patients.
Splavski et al. [14]. 2017 **Locations:** all (central × peripheral) **Design**: retrospective **Cohort:** 243 patients **Outcome:** KPS (at discharge) GOS (at 1 year)	**UVA:** Age Tumor location						When estimating the management outcome of intracranial meningiomas, we propose a simple localization scale dividing the tumors into central and peripheral positions. Central tumor location might be prognostically unfavorable due to the involvement of major neurovascular structures. Among the independent factors predicting favorable tumor response to surgery, the more effective outcome associated with peripheral tumor location and younger age were observed.
	**Centrally located:** Falcine Parasagittal Olfactory groove Clinoidal tumors of the sphenoid ridge Suprasellar/parasellar (*tuberculum sellae*) Cavernous sinus Clival/petroclival Foramen magnum Intraventricular	**Peripherally located:** Convexity Orbital roof Allar and pteryonal sphenoid ridge Middle cranial fossa Cerebellopontine angle Posterior cranial fossa, excluding the vermis					
Meling et al. [8]. 2019 **Locations:** all (SBM × NSBM) **Design:** retrospective + prospective **Cohort:** 1148 patients **Years:** 1990–2002 retrospectively, 2003–2010 prospectively **Outcome measure:** Neurologic status Vital status Surgical mortality OS RFS	Worsening of neurological function was more frequent in SBM (21 vs 13%).						Patients with SBMs had more new-onset neurological deficits and significantly shorter retreatment-free survivals, but this did not adversely affect the overall survival.
Lemée et al. [7]. 2019 **Locations:** all **Design:** retrospective + prospective **Cohort:** 1469 patients **Years:** 1990–2002 retrospectively 2003–2010 prospectively **Outcome measure:** Early complications (hematoma, infection, neurologic worsening, 30-d mortality)	**Postoperative hematoma:** Age RPA (recursive partitioning analysis): Simpson grade, sex, WHO grade, presence of a bone infiltration **Postoperative infection:** NSBM localization RPA: tumor location, preoperative KPS, preoperative clinical status, EOR **Postoperative neurologic worsening:** Postoperative hematoma RPA: postoperative hematoma, EOR **30-day mortality:** Age Postoperative hematoma RPA: postoperative hematoma, EOR, preoperative KPS						Early postoperative complications in meningioma surgery have a negative impact on patient survival and postoperative neurologic status in a disease where survival is usually not limited by the meningioma itself. In this study, we identified risk factors for early postoperative complications; the identification of at-risk populations may help to prevent the occurrence of these risks.
Jenkins et al [5]. 2021 **Locations:** all **Design:** prospective **Cohort**: 345 patients **Years:** 2013–2018 **Outcome:** major AEs (within 3 months; a new focal neurological deficit or grade 3a or higher on the Clavien-Dindo classification scale)	**UVA:** Higher mRS Focal neurological deficit Presence of mental alteration at admission Larger maximum tumor diameter Tumor located in the skull base Higher MCS score **MVA:** Tumor complexity as assessed by score on the MCS **Bivariate analysis—individual variables of MCS:** Tumor size > 4 cm CN manipulation						High tumor complexity is an independent predictor of major AEs following meningioma resection. Preoperative assessment of tumor complexity using the MCS is warranted and can aid communication with patients about AE rates and surgical decision-making. An MCS score of 4 or more was associated with a significant increase in OR for major AEs.
Raman et al.,[12]. 2021 **Locations:** all **Design:** retrospective **Cohort:** 233 patients **Years:** 2007–2019 **Outcome:** mRS scale (favorable × unfavorable dependent, mRS >3)	**UVA:** Supratentorial location Tumor size < 5 cm GTR Comorbidity **MVA:** Tumor size < 3 cm GTR Comorbidity						Tumor size, EOR, age, tumor grade, and medical comorbidities were significantly associated with postoperative outcomes (mRS or RR).

* Milan Complexity Scale—other brain tumors also included

*AE*, adverse events; *CN*, cranial nerve; *EOR*, extent of the resection; *FU*, follow-up; *G*, grade; *GTR*, gross-total resection; *KPS*, Karnofsky performance scale; *MC*, medical complications; *MCS*, Milan complexity scale; *MRC-NPS*, Medical Research Council Neurological Severity Score; *mRS*, modified Rankin scale score; *MVA*, multivariate analysis; *NC*, neurologic complications; *NSBM*, non-skull base meningioma; *NTR*, near-total resection; *OR*, odds ratio; *OS*, overall survival; *p*, points; *QoL*, quality of life; *HRQoL*, health-related QoL; *RFS*, retreatment-free survival; *RPA*, recursive partitioning analysis; *RR*, recurrence rate; *RT*, radiotherapy; SBM, skull base meningiomas; *STR*, subtotal resection; *Sx*, surgery; *UVA*, univariate analysis

## Discussion

Surgical outcome is generally influenced by the patient’s comorbidities, age, presence of neurologic deficit, the size and location of the meningioma, and the experience, surgical strategy, and technique of the surgeon [9]. Individual predictive factors are the subject of several studies [5, 7, 8, 10–15]. and components of a few scoring systems [6, 16–19]. We analyzed the clinical outcome of SBM resection in 552 consecutive patients. KPS remained unchanged or increased in 84.8% and decreased in 15.2% of patients. Morbidity was 13.2% and surgery-related mortality was 1.3%. These results are consistent with the contemporary meningioma series with the reported rates of neurologic morbidity (3.9–13.7%) and mortality (0–5.4%) [5–7]. The preoperative risk factors were analyzed and ROC models estimating the risk of a new neurologic deficit and a decrease in KPS following SBM resection were developed.

In the following paragraphs, we review existing grading systems and compare them with the proposed ROC models. Finally, we provide a brief overview of the risk factors in meningioma surgery.

### Grading systems predicting the functional outcome

The basic principle of medicine is that the benefits of treatment should far outweigh its risks [6]. To facilitate surgical decision-making, different grading systems are often applied in daily practice (e.g., Hunt-Hess classification, Spetzler-Martin classification, GCS) [19, 20, 27, 28]. The practical grading system should be simple, easy to recall, and provide a straightforward preoperative determination [16]. There are five grading systems predicting patient clinical outcomes following meningioma resection: the Levine-Sekhar grading system, the CLASS algorithm, the ABC Surgical Risk Scale, the Predictors of Severe Complications, and the Milan Complexity Scale [6, 16–19]. Selected grading systems with risk factors evaluated and relevant therapeutic recommendations are given in Table 6. Here, we summarize the predictive factors constituting individual scoring systems. Patient-related predictive factors are the patient’s age; [6, 17] comorbidities described by the ASA score; [6] KPS; [17] gravity of symptoms and signs; [6] and the presence of CN III, IV, or VI palsy [19]. Meningioma characteristics predicting the functional outcome are size, [6, 18] location, [6] multiple fossae or posterior fossa involvement, [18, 19] tumor position relative to the central cavity, [16] eloquent area involvement, [18] vessel encasement, [6, 19] CN group involvement, [16] contact with the brainstem, [16] and attachment size [16]. In some scales, previous progression, [6] surgical resection, [6, 16] or EBRT [16, 19] is taken into account. According to the total score achieved, grading scales stratify the patient on the risk of unfavorable clinical outcomes (decrease in KPS and GOS, neurologic deterioration, severe complications, etc.) and recommend therapeutic management.

### Comparison of the ROC models estimating the functional outcome with grading systems

Compared to grading systems, predictive models ensure accurate outcome prediction and patient stratification. Instead of data categorization in the scoring systems, predictive models consider continuous variables (e.g., age, diameter) and their importance. Moreover, categorical data, such as meningioma location, could be more specific (exact location versus dichotomization SBM/nSBM). Although predictive models are often a complicated equation, they could be easily transformed into a surgeon-friendly Excel formula or a mobile phone application.

In this article we propose ROC models estimating the patient’s clinical outcome after SBM resection based on the following variables derived from multivariate stepwise selection analysis: patient age, meningioma size, location and presence of hyperostosis, and dural tail. A major advantage of our ROC models is that they are based on a multicentric study with a relatively large cohort (552 consecutive patients with SBM) compared to scoring systems with one exception based on single institutional retrospective studies with cohorts ranging from 132 to 979 patients [6, 16–19]. In addition, the models are designed for the most at-risk group of patients with surgically demanding SBM. Because the basic predictive variables (patient age, meningioma size, and location) in the studies mentioned are consistent, our models’ considerable convenience is that filling exact patient age, meningioma size, and anatomical location leads to more accurate patient stratification.

Higher age is a risk factor also included in the CLASS algorithm and in the risk of severe complication. While in our ROC model age is considered a continuous variable, in the scoring systems patients are categorized according to age into the risk groups ((≤ 60; 61–71; ≥ 71); (≤ 60; > 70)) [6, 17].

Similarly, the meningioma size included in our ROC models as continuous variable is relatively consistent risk factor considered in 4 out of 5 mentioned scoring system. In the Milan complexity scale, meningioma size ≥ 4.1 cm constitutes a risk factor [18]. Similarly, the ABC surgical risk scale defines three risk groups depending on the meningioma attachment size (< 2 cm; 2–4 cm; > 4 cm) [16]. In the Levine-Sekhar grading system, the risk factor is a presence of multiple fossa involvement [19]. Not quite in line with previous grading systems, the CLASS algorithm consider a higher diameter (≤ 2 cm; 2.1–4 cm; > 4 cm) not only a significant risk factors but also a benefit factor in favor of surgery. The authors’ explanation is the larger the tumor, the greater is the potential benefit for the patient following surgery [6].

The location of the meningioma is an important prognostic factor, but its definition varies significantly among the grading systems. In the CLASS algorithm, the locations are stratified as simple (convexity, lateral and middle sphenoid wing, posterior petrous), moderate (olfactory groove, planum sphenoidale, lateral and paramedian tentorial, parasagittal, intraventricular, cerebellopontine angle, falcine, posterior/lateral foramen magnum, para-sigmoid, and para-transverse sinus locations), and complex (clinoidal, cavernous sinus, tuberculum sellae, medial and incisural tentorial, ventral petrous, petroclival, and anterior/anterolateral foramen magnum) [6]. Adachi et al. in the ABC surgical risk scale define the risk groups by its relation to the central cavity as outside, partial involvement, and inside. The central cavity is the space encircled by the dural entry of CN II–XII [16]. The Milan complexity scale considers a risk factor the posterior fossa and eloquent area involvement [18]. Finally, our ROC models enabled accurate stratification of locations for surgical risk. Petroclival, clival, petrous, and the posterior clinoid process meningiomas were associated with unfavorable clinical outcomes, whereas frontobasal, cerebellopontine angle, and jugular foramen meningiomas with favorable clinical outcomes.

Another risk factors revealed by our ROC model was the presence of dural tail. Dural tail requires an extensive surgical resection with complicated dural repair and a higher risk of CSF leak and wound infection. The implication of dural tail is partly supported by a parameter attachment size from the ABC surgical risk scale, as it reflects both meningioma size and its dural tail [16].

The presence of hyperostosis was a positive prognostic factor in ROC model estimating the risk of a postoperative decrease in KPS. This parameter was statistically significant presumably because of the high prevalence of sphenoid wing and sphenoorbital meningiomas in our series (37.2%). These meningiomas represented 64.7% of tumors with hyperostosis. The presence of hyperostosis was a positive prognostic factor, as it was associated with a high rate of a preoperative neurologic deficit (69.8%) and thus a relatively low potential for clinical deterioration (decrease in KPS of 6.9%), even though another study described the association of hyperostosis with a higher risk of visual impairment [29].

### Risk factors in meningioma surgery

Factors affecting the functional outcome of SBM resection could be divided into patient, tumor, and treatment related [9].

#### Patient-related factors

In line with previous studies, patient age is a well-known prognostic factor of the functional outcome in meningioma surgery [10, 14, 15]. Moreover, older age was associated with higher 30-day mortality, [7] risk of postoperative hematoma, [7] shorter OS, [30] and, in the present study, with a decrease in KPS. It is also a component of the CLASS algorithm by Lee et al. and of the predictors of severe complications by Bartek et al [6, 17].

In the literature, patient’s clinical condition (KPS, modified Rankin scale score, symptoms/signs, and comorbidities) is an important predictor of functional outcome. Bartek et al. documented an association of KPS < 70 with a higher risk of severe complications [17] and Lemée et al. of higher KPS with lower 30-day mortality and infection rate [7]. Similarly, Jenkins et al. reported that the rate of onset of focal neurological deficit or major adverse events was higher in patients with a higher modified Rankin scale score [5]. In contrast, preoperative KPS and neurologic deficit were not statistically significant predictive factors in our study. Inconsistent results were also reported for symptoms and signs. For instance, in the CLASS algorithm, more severe symptoms and signs favor surgery as they could be eventually alleviated [6]. In Meixensberger et al.’s study, CN palsy was associated with an unfavorable outcome, whereas intracranial hypertension, seizure, aphasia, or hemiparesis were associated with a favorable outcome [10]. In the Levine-Sekhar grading system, the CN III, IV, and VI palsies were negative predictive factors [19]. Comorbidities, frequently measured by the ASA score (not evaluated in the present study), are significant predictors of functional outcome in many studies [10, 12]. For instance, Lee et al. incorporated a higher ASA score as a negative predictor in the CLASS algorithm [6].

Patient sex is an inconsistent predictor of functional outcome. Lemée et al., for example, proved its association with neurologic outcome in recursive partitioning analysis, while Jenkins et al. did not [5, 7]. In our cohort, patient sex was not a significant predictor of clinical outcome in univariate or multivariate stepwise selection analysis. However, considering the OS female gender was associated with longer OS after meningioma surgery in French nationwide study [30].

#### Tumor-related factors

The most significant tumor-related risk factor is meningioma size. In the literature, meningioma size or even growth rate, described by several variables (e.g., diameter, dimensions, volume, surface, growth rate mm/year, or cm^3^/year), is considered an important predictor of functional outcome [11, 12]. Meningioma size, variably defined (multiple fossa involvement, [19] diameter, [6, 18] and attachment size [16]), is a component in 4 of 5 of the scoring systems described in this paper. Frequently, meningioma diameter (or attachment size) exceeding 2 or 4 cm is a significant predictor of unfavorable clinical outcomes [5, 6, 16, 18]. In our ROC models, larger meningioma diameter is associated with adverse clinical outcomes.

Another surgically relevant prognostic factor is meningioma location. For statistical analysis, meningioma location is frequently dichotomized (e.g., supra- versus infratentorial, [12] n-SBM versus SBM, [7, 8] and peripheral versus central [14]) the former being associated with a favorable outcome. In the different scoring systems of meningioma location complexity, [6] its relation to the central cavity [16] and multiple [19] or posterior fossa [18] involvement are considered. In contrast, our ROC models enabled accurate stratification of locations for surgical risk. Similarly, Scheitzach et al. reported significant differences in the improvement of the Medical Research Council Neurological Performance and KPS scores among the locations, with olfactory groove and lateral sphenoid wing meningiomas showing the best and foramen magnum meningiomas the worst functional outcomes [13]. According to multivariable analysis in French nationwide study by Champeaux-Depond et al., parasagittal and falx cerebri locations were associated with shorter OS [30].

#### Treatment-related factors

Previous surgical resection and irradiation are well-recognized risk factors associated with high morbidity and complication rates [6, 16, 19, 31, 32]. Considering reoperation for recurrent nSBM, patients with cognitive changes and meningiomas that overlap the middle third of the sagittal plane were at increased risk of complications [31]. In the reoperation of a recurrent SBM, posterior fossa location was significantly associated with complications [32]. In addition, according to multivariable analysis in French nationwide study, redo surgery and radiotherapy for recurrence were predictors of shorter OS [30].

#### Strengths and limitations

The main strengths of this study are its multicentric design and its relatively large cohort of patients. Moreover, all patients treated surgically for a meningioma within the study period were included, which avoids the problem of inclusion bias. Surgeries were performed in six neurosurgical departments within a geographically well-defined area with equal patient access to health care, thereby minimizing the risk of selection bias. A few Czech neurosurgical departments did not participate; therefore, the study does not meet the criterion of a national study. The major limitations are the retrospective nature of parts of the study, the relatively short postoperative follow-up, and the limited number of patients with SBM in rare locations (e.g., jugular foramen, tuberculum sellae, clivus).

## Conclusion

An evidence-based therapeutic approach should be based on known risk factors, scoring systems, and predictive models. Predictive models allow rapid assessment of surgical risk, which could be compared to the natural history, SRS, EBRT, and combined approach, their efficacy, and complications. In addition to reviewing the basic literature, this article provides ROC models estimating the functional outcome of SBM resection based on patient age, meningioma size and location, and the presence of hyperostosis and dural tail. The next step is to validate the ROC models on a larger prospective patients’ cohort.

### Supplementary information

ESM 1

ESM 2
